# Morphological and dimensional variations of the pterygoid hamulus: A CBCT-based cross-sectional study

**DOI:** 10.6026/973206300221477

**Published:** 2026-03-31

**Authors:** Vijayalakshmi Gachinamath, Praveenkumar Ramdurg, S Naveen, K Mohammed Fazil, ZebaAfroz I Shaikh, C Jyoti, Harshita K Arabbi, Mehul A Shah

**Affiliations:** 1 Department of Oral Medicine and Radiology, PMNM Dental College and Hospital, Bagalkot- 587103, Karnataka, India; 2 Department of Public Health Dentistry, SDM College of Dental Sciences and Hospital, Shri Dharmasthala Manjunatheshwara University, Dharwad, Karnataka, India; 3 Department of Public Health Dentistry, Government Dental College and Hospital, Chhatrapati Sambhajinagar (Aurangabad), Maharashtra, India

**Keywords:** Cone-beam computed tomography (CBCT), craniofacial anatomy, hamular syndrome, pterygoid hamulus (PH), variations

## Abstract

Variations in the morphology of the pterygoid hamulus (PH) can lead to oropharyngeal pain, prosthodontic challenges and surgical complications, yet precise *in vivo* evaluations are limited. Therefore, it is of interest to assess the morphometric variations of the pterygoid hamulus (PH) in the Bagalkot population using Cone-Beam Computed Tomography (CBCT). CBCT scans of 65 adults (46 males, 19 females; mean age 39.5 years) were retrospectively analyzed, focusing on PH dimensions and shape. Results indicated that females exhibited slightly higher mean PH length, width and angulation than males, with width showing greater variability in females. Slender morphology was predominant bilaterally, followed by triangular types in both genders. The findings advance knowledge by demonstrating the utility of CBCT for accurate PH evaluation, supporting diagnosis of hamular-related pain and aiding in prosthodontic and maxillofacial procedures.

## Background:

The pterygoid hamulus (PH) is a delicate, hook-shaped bony projection arising from the inferior extremity of the medial pterygoid plate of the sphenoid bone. Anatomically, it is situated posterior to the maxillary alveolar arch and inferior to the posterior border of the hard palate, occupying a strategic position within the nasopharyngeal and oropharyngeal region [1]. Despite its small size, the PH plays a crucial functional role in the craniofacial complex. Functionally, the PH acts as a pulley for the tendon of the tensor veli palatini muscle, facilitating its redirection toward the palatine aponeurosis [2]. Additionally, it provides attachment to fibres of the levator veli palatini and superior pharyngeal constrictor muscles, contributing significantly to velopharyngeal closure, swallowing, speech articulation and regulation of middle ear pressure through Eustachian tube opening [3]. Any alteration in the morphology or orientation of the PH can therefore disrupt these essential physiological processes. Considerable anatomical variability of the PH has been documented in the literature. Morphometric differences in length, width, angulation and shape have been observed across populations, age groups and genders [4]. Such variations may influence adjacent soft tissues, including the palatal mucosa and pharyngeal wall, potentially resulting in localized irritation or functional impairment [5]. Abnormal elongation, thickening, or angulation of the PH has been associated with pterygoid hamulus bursitis, also referred to as hamular notch syndrome. This condition is characterized by chronic palatal pain, dysphagia, foreign body sensation, otalgia and pain radiating to the temporal or mandibular regions, often leading to misdiagnosis. Conventional radiographic techniques offer limited visualization of the PH due to superimposition and distortion [6]. The advent of Cone-Beam Computed Tomography (CBCT) has revolutionized maxillofacial imaging by enabling high-resolution, three-dimensional assessment of craniofacial structures with uniform magnification and comparatively lower radiation exposure than conventional computed tomography. CBCT has proven particularly valuable in the evaluation of small anatomical structures such as the PH, allowing accurate morphometric analysis *in vivo* [7]. Accurate knowledge of PH morphology is essential in various clinical scenarios, including Le Fort I osteotomy, pterygoid implant placement, maxillary posterior surgical procedures, prosthodontic rehabilitation and evaluation of obstructive sleep apnea, where PH length and inclination may influence soft palate dynamics [8]. Establishing normative CBCT-based reference values for PH morphology can therefore enhance preoperative planning, minimize iatrogenic complications and improve diagnostic accuracy in patients presenting with unexplained orofacial pain [9]. Therefore, it is of interest to determine the length, width, inclination and shape of the pterygoid hamulus in the Bagalkot population using CBCT imaging, providing insights for accurate clinical interpretation and enhancing oro-facial function and pathology diagnosis.

## Materials and Methods:

This cross-sectional study was conducted using Cone-Beam Computed Tomography (CBCT) images obtained from patients who reported to the Department of Oral Medicine and Radiology, P.M.N.M. Dental College and Hospital, Bagalkot. All available full-volume CBCT scans acquired over a one-year duration were retrospectively reviewed.

## Inclusion criteria:

[1] Patients aged ≥20 years

[2] CBCT scans showing clearly identifiable pterygoid hamulus bilaterally

[3] Symmetrical and undistorted images suitable for morphometric analysis

## Exclusion criteria:

[1] History of trauma or fractures involving the maxillary posterior region

[2] Pathological lesions (cysts, tumors, infections) affecting the pterygoid or palatal region

[3] Developmental anomalies or previous surgical intervention involving the pterygoid plates

[4] CBCT scans exhibiting blurring, motion artifacts, truncation, or insufficient image quality resulting in incomplete or unclear visualization of the pterygoid hamulus

## Measurements:

[1] Measurements were performed using CBCT software tools and included:

[2] Length: Measured from the junction of the medial pterygoid plate to the tip of the PH.

[3] Width: Measured at the thickest portion in the coronal plane.

[4] Inclination: Assessed in the coronal (medial/lateral) planes.

[5] Shape: Categorized as slender or triangular.

All parameters were assessed bilaterally. Measurements were independently evaluated by certified Oral and Maxillofacial Radiologists.

## Results:

The descriptive statistics comparing the mean morphometric measurements between male and female participants for right and left side parameters, including length, width, and angulation is shown in Table 1 (see PDF) and Figure 1 (see PDF). On the right side, the mean length was 4.8413 in males and slightly higher in females at 5.0684. Similarly, the mean right-side width measured 1.2739 in males compared to 1.3211 in females, indicating marginally greater transverse dimensions in females. The mean right-side angulation was 21.95 in males and 22.7526 in females, demonstrating a small but consistent increase in angular measurement among female subjects. On the left side, the mean length was 4.637 in males and 4.9684 in females, again showing slightly higher values in females. The mean left-side width was 1.287 in males and notably higher in females at 1.5158, reflecting a more pronounced difference compared to the right side. The most substantial variation was observed in left-side angulation, with males exhibiting a mean value of 24.5652 and females showing a higher mean of 27.2105. The mean angulation obtained is 22.1 degree on right side and 25.3 degree on left side with p value more than 0.5 showing no significant difference. The study sample comprised 65 participants with a mean age of 39.5 years, including 46 males and 19 females. Descriptive analysis of pterygoid hamulus (PH) morphology revealed minor gender-based differences in length, width, angulation and shape distribution. Categorical assessment of PH shape revealed that, on the right side, a slender morphology was predominant in males (73.9%), followed by triangular morphology (26.1%). Among females, 57.9% exhibited a slender shape, while 42.1% demonstrated a triangular shape. On the left side, slender morphology predominated in both males (76.1%) and females (73.7%). Triangular morphology was observed in 23.9% of males and 26.3% of females (Table 2 - see PDF). Females demonstrated a slightly greater mean right PH length (M = 5.07, SD = 1.40) compared to males (M = 4.84, SD = 1.66). A similar pattern was observed for left PH length, with females showing a mean of 4.97 (SD = 1.55) and males a mean of 4.64 (SD = 1.76) with p value more than 0.5 which was not significant. Regarding PH width, the mean right PH width in females was 1.32 (SD = 0.59), marginally higher than in males (M = 1.27, SD = 0.38). On the left side, females exhibited greater variability in width (M = 1.52, SD = 1.39) compared to males (M = 1.29, SD = 0.56) with p value more than 0.5 which was not significant. Analysis of PH angulation showed that females had a mean angulation of 22.75°C (SD = 6.79) on the right and 27.21deg;C (SD = 11.47) on the left. Corresponding values in males were 21.95°C (SD = 9.94) for the right side and 24.57°C (SD = 12.32) for the left side. With p value more than 0.5 which was not significant on the right side, 73.9% of males exhibited a slender pterygoid hamulus, while 26.1% showed a triangular morphology. In contrast, among females, 57.9% demonstrated a slender shape and 42.1% a triangular shape (Figure 2 - see PDF). On the left side, 76.4% of males had a slender morphology, whereas 23.9% exhibited a triangular shape. Similarly, in females, 73.4% showed a slender morphology and 26.3% a triangular morphology (Figure 3 - see PDF). Statistical analysis revealed a significant correlation in the distribution of pterygoid hamulus morphology between sides and genders, with a p-value < 0.01.

## Discussion:

The present study evaluated the morphometric characteristics of the pterygoid hamulus using CBCT and identified minor gender-related variations in length, width, angulation and shape. Overall, the findings suggest substantial individual variability with limited sexual dimorphism. The mean PH length observed in this study corresponds well with previously reported adult values ranging from 4 to 7 mm. slightly greater mean values in females have been documented in some studies, although these differences are generally considered clinically insignificant and may reflect individual craniofacial growth patterns rather than true sexual dimorphism [9]. PH width demonstrated greater variability, particularly in females. Similar findings have been reported in CBCT-based studies emphasizing the anatomical diversity of this structure. Increased width or irregular morphology may predispose individuals to soft tissue irritation, contributing to symptoms such as palatal pain or dysphagia [10]. Angulation of the PH has received increasing attention due to its role in hamular bursitis. Excessive angulation may alter the biomechanics of the tensor veli palatini tendon, increasing friction and local inflammation. However, the wide overlap of angulation values observed suggests that angulation alone may not be a reliable predictor of symptoms [11]. The predominance of the slender PH morphology observed aligns with earlier anatomical and radiological studies. The triangular variant, although less frequent, is considered clinically significant due to its association with palatal pain syndromes and localized inflammation [12]. CBCT remains a valuable imaging modality for accurate assessment of PH morphology, enabling clinicians to differentiate normal anatomical variations from clinically relevant abnormalities [13].

## Conclusion:

The pterygoid hamulus exhibits considerable morphological variability with minimal gender-related differences. CBCT provides a reliable and accurate method for evaluating PH morphology. Recognition of these anatomical variations is essential for improved diagnosis, treatment planning and prevention of complications related to hamular pathology.
